# The Intersubjective Nature of Play Development and Its Role in Child Psychoanalytic Psychotherapy

**DOI:** 10.3389/fpsyg.2016.01783

**Published:** 2016-11-15

**Authors:** Vera R. R. Ramires

**Affiliations:** Graduate Program in Clinical Psychology, Universidade do Vale do Rio dos Sinos, UnisinosPorto Alegre, Brazil

**Keywords:** play, children, psychoanalytic psychotherapy, psychoanalysis

Playing is a vital activity in childhood. It is important to psychological development, in both cognitive, emotional, and social dimensions. It has a core role to the young child's mental health. In the clinical setting, play has had an important function, enabling a way of communicating with the child, and a privileged way for creation of meanings and expression of feelings, affects, fears, angry, difficulties, etc. In this article, I highlight the intersubjective nature of play development, its impediments and its crucial role in child psychoanalytic psychotherapy.

## First developments in child psychoanalysis and the use of play

One of the pioneers of psychoanalysis with children was Melanie Klein. She introduced the use of play in the analytical process. More than that, she developed a theory which describes the importance of playing for ego development and the interplay between internal and external reality (Klein, 1930, 1932, 1955). According to Klein, children's object relations begin almost at birth, and arise with the first breastfeeding experience (Klein, 1955). The way the child experiences the external world is constantly influenced by—and influences—the internal world which is being developed. Envy and destructive impulses have an important role in her theory, especially primitive sadism (Klein, 1932), and the ego development occurs on the basis of these intense drives and conflicts.

The process of symbol-formation, which becomes possible when frustrating experiences and destructive impulses were not prominent, is a core achievement for ego development and mental health. Playing becomes possible when the child is able to symbolize, that is, displace his/her emotions, fears, and anxieties to other objects in the external world, not the primary objects (Klein, 1930). When playing, the child feels relief because he/she transfers fantasies, anger, anxiety and guilt to other objects that are not his/her primary caregivers. Klein stated that play and dreams share analogous means of representation, and in both there is a form of wish fulfillment. Play's specific content is identical to the core of the child's masturbatory fantasies, and one of its main functions would be to provide a discharge for these fantasies. The role of the analyst would be to interpret the fantasies and anxieties underlying the play or the inhibitions to play when they are present.

Winnicott (1971) followed Klein's steps in object relations approach, but he introduced new and important ideas to the understanding of child's play. According to Winnicott, we must think about playing as a thing in and of itself, overcoming the concepts of instinct sublimation and play as performing masturbatory fantasies. He postulated the existence of a potential space between the baby and the mother, where the play takes place. In early childhood, this intermediate area is necessary to start the relationship between the child and the world. Winnicott described the transitional phenomena and the importance of illusion for the child in this process. A good enough mother is able to adapt almost completely to the initial needs of her baby. This gives the baby the illusion that there is an external reality corresponding to his/her own ability to create. It is essential that the mother survives to the baby's aggression to establish his illusory omnipotence. Besides, this almost complete adaptation should gradually decrease, and the following disappointment creates an intermediate area of experience, which is the area of play and, later, the arts, religion, imagination, and scientific work.

In sum, playing is a creative experience, which makes possible a sense of continuity in space and time for the baby and the discovery of self (Winnicott, 1971). Psychotherapy takes place in the overlap of two ludic areas, of the patient and of the therapist. Therefore, if the child cannot play, “then the work done by the therapist is directed towards bringing the patient from a state of not being able to play into a state of being able to play” (p. 38).

As we can see, the importance of the relationship between a child who plays and another person (the mother, a caregiver or the therapist, in the clinical context) soon began to be highlighted in psychoanalytical approaches. This leads us to consider the caregivers' role in developing the child's ability to play, as formulated by contributions from theoreticians of attachment and reflective function.

## Contributions from contemporary theorists of attachment approach

Contemporary authors who followed the tradition of object relations theory and the latest contributions of attachment theory argue that the child's attachment bond with his/her parents serves mainly as self-organization and as an emotional regulation system (Fonagy et al., 2002). In this scenario, play has “a pivotal role in the developing of thinking as well as emotional experience, and particularly in their integration” (Fonagy and Target, 1996, p. 220).

According to Fonagy and Target (1996), the very young children (2–3 years old) present a dual character regarding their sense of inner reality. In the “psychic equivalence mode,” ideas are direct replicas of reality, so that they are always true. They are not felt to be representations. For a small child, his/her inner experience is equivalent to and thus mirrors the external reality. In a similar manner, the other has the same experience. On the other hand, in the “pretend mode” ideas are felt to be representational, but their correspondence with reality is not examined.

In normal development (4–5 years old), the child integrates these two modes to achieve the stage of “mentalization,” or “reflective mode,” when mental states are experienced as representations. Furthermore, inner and outer reality can then be seen as linked (Fonagy and Target, 1996; Target and Fonagy, 1996; Fonagy et al., 2002). Mentalization “comes about the child's experience of his mental states being reflected on, prototypically through experience of secure play with a parent or older child, which facilitates integration of the pretend and the psychic equivalence modes, through an interpersonal process that is perhaps an elaboration of the complex mirroring of the infant by the caregiver” (Fonagy et al., 2002, p. 57). In order to achieve this integration, three things are needed: child's mental states (feelings and thoughts) need to be represented in the object's mind; the frame represented by the object's perspective, generally reality-oriented; an adult or older child who “plays along,” so that the child sees his/her fantasies or ideas represented in other's mind, re-introjects this and uses it as a representation of his/her own thinking (Fonagy and Target, 1996).

Tessier et al. (2016) found empirical evidence to support these conceptions. In a longitudinal study with sexually abused and non-abused children, they analyzed if children's capacity to engage in pretend play, to symbolize and to make play narratives was associated with later reflective functioning, and if play mediated early child sexual abuse and later mentalization. They found that children's capacity to elaborate and conclude play narratives predicted later mentalizing abilities. Interestingly, play predicted later mentalization regarding others, but not regarding self. As an explanation the authors stated that mentalization regarding self can be expected to be more closely related to the primary caregiver's interest in the child's subjectivity.

## Play in diagnosis and therapeutic process

As we can see, the way of considering play in the psychoanalytic psychotherapy has changed since Klein's pioneer work. Despite the richness of her contributions, her theory maybe assumes a highly sophisticated mental apparatus from the beginning of psychological development, with too much emphasis on the destructive impulses and early oedipal and sexual fantasies. According to Klein, playing and its development seems like an individual activity, or basically depends on the individual and the intensity of his/her destructive impulses. The therapist's task would be to understand and to interpret the play's content and meaning to the unaware child.

Winnicott, on the other hand, values play beyond the need to discharge impulses and communicate conflicts. He stresses that the psychoanalyst must look at the playing child, moving on from the play content, exclusively.

To sum up, playing is an interpersonal activity, since its beginning. It involves the symbolic transformation of the reality and it requires the presence of the other, and a view to the other's mind to occur (Target and Fonagy, 1996). Moreover, even if children under 4 years old are able to symbolize, they do not have symbols for their thoughts, which means that they do not treat their thoughts as symbolic, representing rather than directly reflecting the objective reality. For the authors, a symbol would be a representation of a mental representation. Thus, children need the other (their caregivers in normal development or their therapist in a clinical setting) in order to achieve representation and understanding of mental states and inner reality.

Notwithstanding the importance of play in child psychotherapy, as child psychotherapists we know many children who cannot play at all, or show a disorganized and chaotic play. I argue that with some children the work to be done is to develop their capacity to play, in the way described by Slade (1994), which matches with contemporary attachment and the reflective functioning approach. In terms of psychopathology, play being present and having a symbolic meaning would require that the conscious and the unconscious were clearly established and consolidated, and experiences that are reference had suffered repression. When a child lives in a disorganized emotional universe, experiencing the inner life as diffuse and unintegrated, not only his/her capacity to play will be impaired, but the use of verbal interpretations will be disruptive and lead to denial and further disorganization. Besides, when the therapist makes verbal interpretations, this places him/herself outside the play, what is quite different from playing with the child, and assumes that the child has the ability to reflect upon the play and his/her inner life.

When working with young or very disturbed children, the therapist must help to develop their capacity to play. By developing children's capacity to play, therapists will help them to create meaning, more than uncovering meaning (Slade, 1994). In other words, by means of play, children will discover what they feel, think and want, and what others feel and believe. As the caregiver would have done whenever possible, therapist and child build narratives about child's psychological reality. Slade highlights some functions of the play, which I consider core functions in the clinical setting: the development of a narrative, the integration of affect into the narrative, the contextualization of meaning making within an object relationship and the development of reflective self-function. Again, this process will only become possible and the child will represent internal experiences by playing only if these experiences are first made real by another's recognition of them.

All this suggests that play has a pivotal role in the whole therapeutic process, including the assessment period. The level in which the child operates regarding the sense of inner reality (equivalence mode, pretend mode or reflective and integrated mode) provides the therapist with valuable information when he/she analyzes play characteristics (or its absence). Furthermore, play has various dimensions, like affective, cognitive, narrative and developmental components (Chazan, 2012), beyond its symbolic quality and psychodynamic aspects. All these parameters can help the clinician in children diagnosis and treatment planning. In the same way, these parameters make possible to analyze the therapeutic process, its progress and retreats, and even the treatment's outcomes.

In conclusion: (1) playing is an essentially intersubjective activity; (2) it enables the construction/organization of internal and external realities; (3) the ability to “play” with these internal and external realities, in the sense proposed by Target and Fonagy (1996), leads to greater autonomy, freedom and robustness of the child's psychological organization; (4) when playing is not possible, it needs to be developed, either within the family, educational or clinical context. The approach discussed here may provide aid to interventions for families with very young children, interventions in the nursery and pre-school context and, of course, in the clinical setting.

## Author contributions

The author confirms being the sole contributor of this work and approved it for publication.

## Funding

National Council of Technological and Scientific Development (CNPq)—Grants 471358/2014-2 and 311235/2014-0.

### Conflict of interest statement

The author declares that the research was conducted in the absence of any commercial or financial relationships that could be construed as a potential conflict of interest.
